# Development and testing of the aerial porter exoskeleton

**DOI:** 10.1017/wtc.2021.18

**Published:** 2022-01-07

**Authors:** W. Brandon Martin, Alexander Boehler, Kevin W. Hollander, Darren Kinney, Joseph K. Hitt, Jay Kudva, Thomas G. Sugar

**Affiliations:** 1 Ira A. Fulton Schools of Engineering, Arizona State University, Mesa, Arizona, USA; 2 Augspurger Komm Engineering, Phoenix, Arizona, USA; 3 GoX Studio, Phoenix, Arizona, USA; 4 NextGen Aeronautics, Inc., Torrance, California, USA

**Keywords:** exoskeleton, hip assist, powered exoskeleton, push assistance, lift assistance, lifting, pushing

## Abstract

Back pain is one of the largest drivers of workplace injury and lost productivity in industries around the world. Back injuries were one of the leading reasons in resulting in days away from work at 38.5% across all occupations, increasing for manual laborers to 43%. While the cause of the back pain can vary across occupations, for materiel movers it is often caused from repetitive poor lifting. To reduce the issues, the Aerial Porter Exoskeleton (APEx) was created. The APEx uses a hip-mounted, powered exoskeleton attached to an adjustable vest. An onboard computer calculates the configuration of the user to determine when to activate. Lift form is assisted by using a novel lumbar brace mounted on the sides of the hips. Properly worn, the APEx holds the user upright while providing additional hip torque through a lift. This was tested by having participants complete a lifting test with the exoskeleton worn in the “on” configuration compared with the exoskeleton not worn. The APEx has been shown to deliver 30 Nm of torque in lab testing. The activity recognition algorithm has also been shown to be accurate in 95% of tested conditions. When worn by subjects, testing has shown average peak reductions of 14.9% BPM, 8% in VO2 consumption, and an 8% change in perceived effort favoring the APEx.

## Introduction

Back pain is one of the largest drivers of workplace injury and lost productivity in industries around the world. According to the Bureau of Labor Statistics, back injuries were one of the leading reasons in resulting in days away from work at 38.5% across all occupations (U.S. Bureau of Labor Statistics, 2018). This statistic is increased for manual laborers to 43%. While the cause of the back pain can vary across occupations, for materiel movers it is often caused from repetitive poor lifting. The United States Air Force (USAF) is not exempt from this and noticed significant issues with their workforce, specifically aerial porters. These individuals are ultimately responsible for loading and unloading cargo from planes at bases across the world. Their operational tempo requires that this be done in a quick and efficient manner to accomplish their mission and enable global operations. The act of physically loading pallets onto aircraft is challenging. Pallets on their own are large and can often be irregular. Paired with massive weight in the thousands of pounds, a team is often needed to maneuver the cargo into position. This involves strong pushes putting strain throughout the spine. This is not their only responsibility. Aerial porters have an additional duty of handling bags and smaller cargo to get them to a site where they can be palletized. In austere locations, this can mean hand-carrying large, heavy bags over long distances multiple times a day, to say nothing of actually arranging them. For these reasons, the USAF sought a solution that would alleviate fatigue and lower back pain during logistics operations and allow their workforce to remain healthy without injury. Multiple companies and universities are developing solutions to assist in hip motion and stabilize the back with the goal of reducing workplace back injuries (Hollander et al., 2014a, 2014b; Kerestes et al., 2014; Asbeck et al., 2015; Seo et al., 2017). To assist the lifting motion, the developed devices must follow the general rules of thumb: be lightweight, energy efficient, and human conformable. For daily operations, normal human gait such as walking and jogging must also remain unimpeded. These are the basic guidelines to help inform the design to create a wearable device that causes a net benefit to the user. For airfield operations, the guidelines are more demanding: last a full day (8 hr), be compact, and require little to no human involvement. It is with this in mind that the Aerial Porter Exoskeleton (APEx) was created.

### Passive versus Powered Systems

There are many exoskeletons on the market that aim to help warehouse workers accomplish daily lifting tasks while protecting their back. Many of them are commercially available today. Generally, these, and other exoskeletons, fall into one of two categories: passive or powered. Passive exoskeletons operate by capturing energy in one movement and storing it for later, more advantageous, release. For lower body exoskeletons, this is often done through a spring mechanism, using motion with gravity to “charge” the spring and discharging through the standing motion. Such exoskeletons, properly used, have been shown to reduce metabolic costs as well as allow the user to complete more “good” lifts over a longer period (Schmalz et al., 2019). Passive systems, lacking a motor and the attendant electronics, are often lighter and more affordable than similar powered systems. Notably, they are incapable of providing additional power when needed, only what is stored through the user’s actions can be released by the system. Powered exoskeletons, on the other hand, can assist more effectively in heavy operations. These systems can provide extra torque to the hips without requiring the user to “charge” the device, allowing for a stronger overall action. Creating these devices is often more complicated than a passive system. For a powered system to be properly implemented, it must anticipate a user’s intention and respond accordingly, so as to appear physically invisible to the user. Without this invisibility, the user often feels as though they have an added weight, reducing the efficacy of the device, sometimes drastically physically (Gregorczyk et al., 2010; Young et al., 2017; Kim et al., 2018a, 2018b; Sado et al., 2018, 2019) and mentally (Bequette et al., 2020). The APEx was designed to be a powered system while taking these limitations in mind. After creating initial design goals, representatives in the aerial porter field were interviewed for their insights on what would be useful. They returned with two key observations. One, nothing can be worn at the foot or shoe level. The workers are often moving through tight areas with irregular flooring. Two, when pushing, turning to place your back on the object is considered to be easier since the lumbar region is supported. While it is more comfortable, it is more dangerous with a reduction of control on the object. Our main design goals included the following:

1. The system would not impede motion when walking, jogging, or running.
2. The system would aid lifting heavy objects by assisting hip extension.
3. The system would aid pushing heavy objects by assisting hip extension.
4. The system would be disengaged during all other motions to allow truly free motion.
5. Extra degrees of freedom were added to the actuator unit to allow for hip abduction/adduction.

## Methods

### Mechanical Design

#### Actuation method

**Figure 1.**
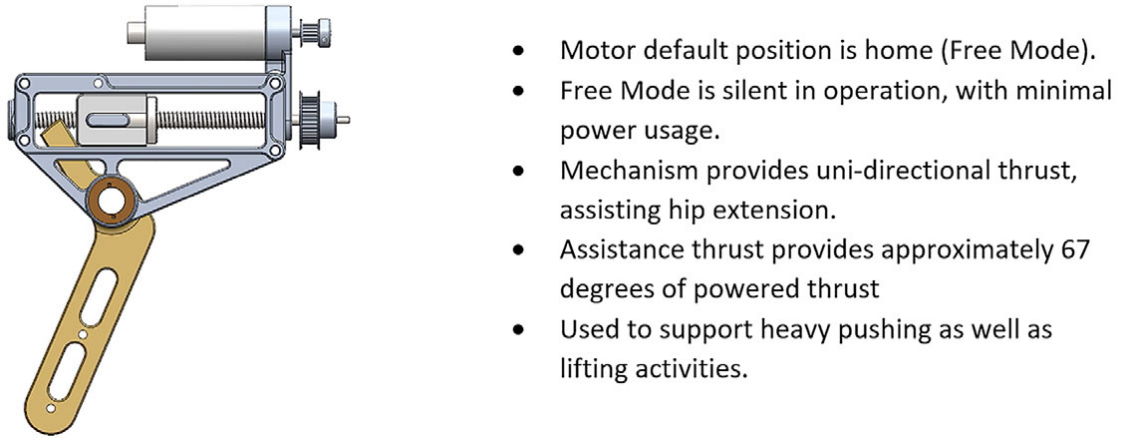
A drive block is used to apply a thrust to the lever arm which is attached to the hip pad. The actuator as shown is worn on the left hip, with the bronze stem resting on the top of the users left thigh. The block is extending the user’s leg about the hip by driving toward the viewer’s left.

To meet the demands of this project, the APEx needed a new type of actuating mechanism. The core of the APEx’s mechanism is similar to the HeSA exoskeleton created by K.W.H. and T.G.S. The HeSA delivered hip torque through a lever arm that rotated in the same axis as the weare’s hip (sagittal plane). At the top of the lever arm, a fork was designed that straddled a ball screw. On the ball screw, surrounding the fork, there was a block that moved back and forth as the ball screw moved. As the block moved, so did the fork and by extension, the lever arm. This had the effect of delivering torque in either direction, assisting both hip flexion and extension: upswing of the leg in the swing phase of gait and leg extension in squat recovery. While the method proved effective, it was limited in speed and unsuitable for the USAF’s needs. The APEx’s iteration removed the “surrounding” nature of the actuating block, instead placing the block behind the fork. This allowed the block to move out of the way when not in use, rendering it invisible to the user’s motion. When in use, the block could deliver a powerful push to the fork, assisting the user in squat recovery and pushing. Specifications of this design are listed in Table 1. The basic outline illustrating the mechanism can be seen in Figure 1. This new design is patent pending.

**Table 1.** The APEx device provides unidirectional torque support for hip extension to each hip, see Figures 2 and 3

APEx metrics indicating output and mass	
Peak torque for hip extension per leg	30 Nm
Total device weight without battery	3.26 kg
Battery weight, 36 V 72 Whr	0.68 kg

Abbreviation: APEx, Aerial Porter Exoskeleton.

#### Human conformity

**Figure 2.**
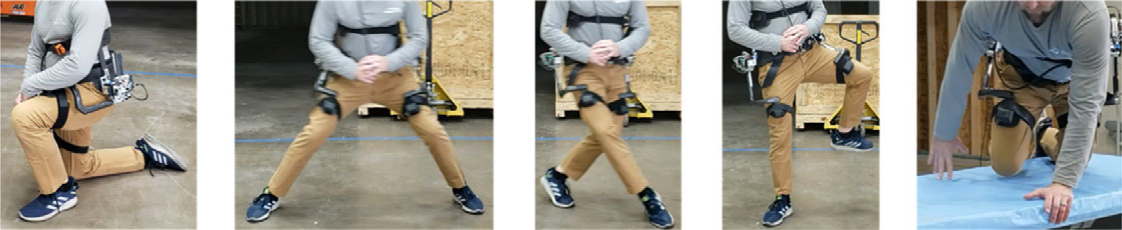
With the motors turned off in a free mode, the user can knee, take wide and cross steps, rotate the hip and crawl.

**Figure 3.**
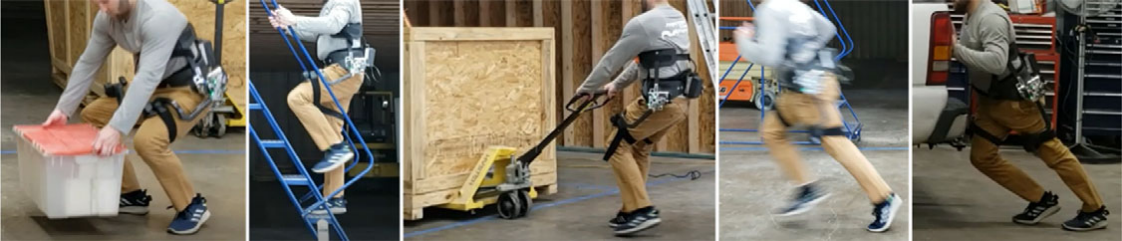
In an active mode, the hip extension is assisted when lifting a box, going up stairs, and pushing a heavy object. During modes such as jogging or running, the block moves out of the way and the hip actuator is in free mode.

**Figure 4.**
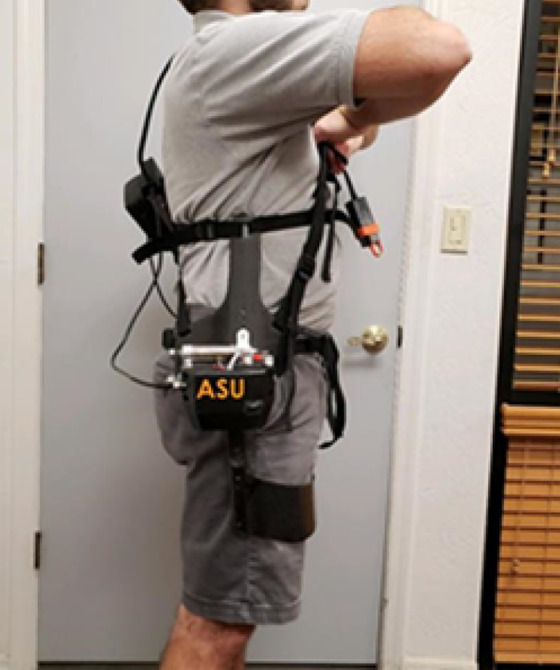
Lumbar plates are used to resist the 30 Nm of torque supplied per motor and keep the back straight during lifting and pushing activities.

A large challenge existed in creating a solution that allowed the APEx to anchor itself solidly to the user while remaining light and compact enough to hit design goals and not negatively affect the user (Pons, 2008; Aliman et al., 2017). Iteration on the design occurred in three major forms. The first form saw the mechanism attached to the hip with a semirigid plate and a flexible belt system. This allowed for rigid holding at the hips and a relatively simplistic means of donning. When tested though, the brace rotated up and into the user, causing discomfort and a large amount of lost delivered torque. The second form attempted to improve on this by creating a rigid back brace to take advantage of the larger surface area. This brace was adjustable to accommodate different hip widths and allow for a tighter overall grip on the subject. Testing revealed that it did indeed hold better, however it was still too loose, bending into the user and causing discomfort in the stomach and back. The third solution was to run braces up the side of the body rather than up the back. These lumbar plates are a unique design element. This helped to avoid the discomfort often reported with other exoskeletons and kept weight down. The side brace works by anchoring at the top just below the ribs, and around the waist. This arrangement had the added benefit of keeping the user’s back straight during lifting activities without physically pressing into the lumbar region, while providing large unhindered, unassisted motion capabilities (Figure 2, 3). This pelvic plate design is patent pending (Figure 4).

### Software Design

As with any powered exoskeleton, the control schema must allow for different movement activities. There are different input possibilities ranging from IMUs (Kazerooni et al., 2005) to EEGs (Kilicarslan et al., 2013) to EMGs (Yousif et al., 2019) to force sensors (Kazerooni et al., 2006) to series elastic actuation (Dollar and Herr, 2008; Pons, 2008). A poorly executed system will cause the system to move during the wrong motions, potentially causing injury (Browning et al., 2007). To control the APEx, a redundant activity recognition algorithm (ARA) system was created with a basic model and an AI trained model. Both models operate on the white-list principle, in which the system will only activate when it is certain that a lift or push is taking place. For all others it will not engage. The basic activity recognition uses IMUs fitted in the control chip at the hip (one on each side), and rotation sensors about the axis of rotation (one on each side). Using anthropomorphic data available from the U.S Army Natick Center (Gordon et al., 2014) a basic kinematic model was created with measurements of different data were normalized to thigh length. It was also assumed that the foot remained horizontal in any motion. With these measurements, the center of gravity (COG) was determined. Live tests indicated that situations that required assistance placed the COG in front of the heel of our model. This held true for all tested parameters including walking, sitting, climbing into a driver’s seat, and pushing a cart downhill. Using this model, a basic ARA was created. The advanced ARA (AARA) used a machine learning-trained model informed by IMUs placed at the top of the hip brace, the side, the lever arm, and the back. Then, motion data was captured while wearing the APEx to identify the signatures of squatting, sitting, pushing, walking, jogging, climbing stairs, and vehicle entry/exit. This was then fed into a machine learning algorithm that a set of parameters for the internal control board to report on. Tests of the AARA showed a 95% success rate at identifying the activity.

## Experiment

### Participants

Thirteen participants with no history of injury, neurological disease, back pain, and with an unaffected natural walking gait were selected for this study. All tests were conducted under the protocol as per the guidelines of the Institutional Review Board at Arizona State University. Data was collected at Travis Air Force Base in California. Before any measurements were taken, participants were informed of the testing protocol and given a consent form. If consent was given, participation continued, if not, the test was terminated and participants were free to leave. There was no payment for completion of the experiment.

### Test Protocol

Before beginning, participants were fitted with a GoX Ergo Kit (GoX Studio, 2020) to measure heart rate and VO2 consumption. Measurements of their resting heart rate, height, weight, and age were also taken. Participants were then placed in experiment format A or B. Format A had participants using the exoskeleton in the powered-on configuration for the experiment procedure first, and without the exoskeleton worn for the second run. Format B was the opposite. The test was controlled so that two participants completed format A, and one completed format B. For the tests, participants were asked to begin by standing behind a weighted (22.7 kg) container. When told to start, participants would then lift the box to a comfortable height and carry it 6.1 m. Participants would then set the box down, walk behind it, and turn, facing the original direction to repeat the process. This task of moving a box 6.1 m and then moving the box back to the original position was considered one set. Participants were given 30 s to complete each set, marked with an audible chime. Any time remaining within the 30 s after placing the box down was used as a rest period. Participants completed 30 sets in total. Participants were then asked to give their rate of perceived exertion (RPE) on a 0–20 scale. Once complete, participants were given 30 min to rest before repeating the process in their predetermined format.

### Post Processing

Data collected from the test were then used to calculate the average and peak heart rates and VO2 consumption. A starting heart rate was determined as the average of four samples taken before the start of the test. The tested values were chosen as an average of four samples within the last 4 min of the test. The percent change was then calculated.

## Results

**Figure 5.**
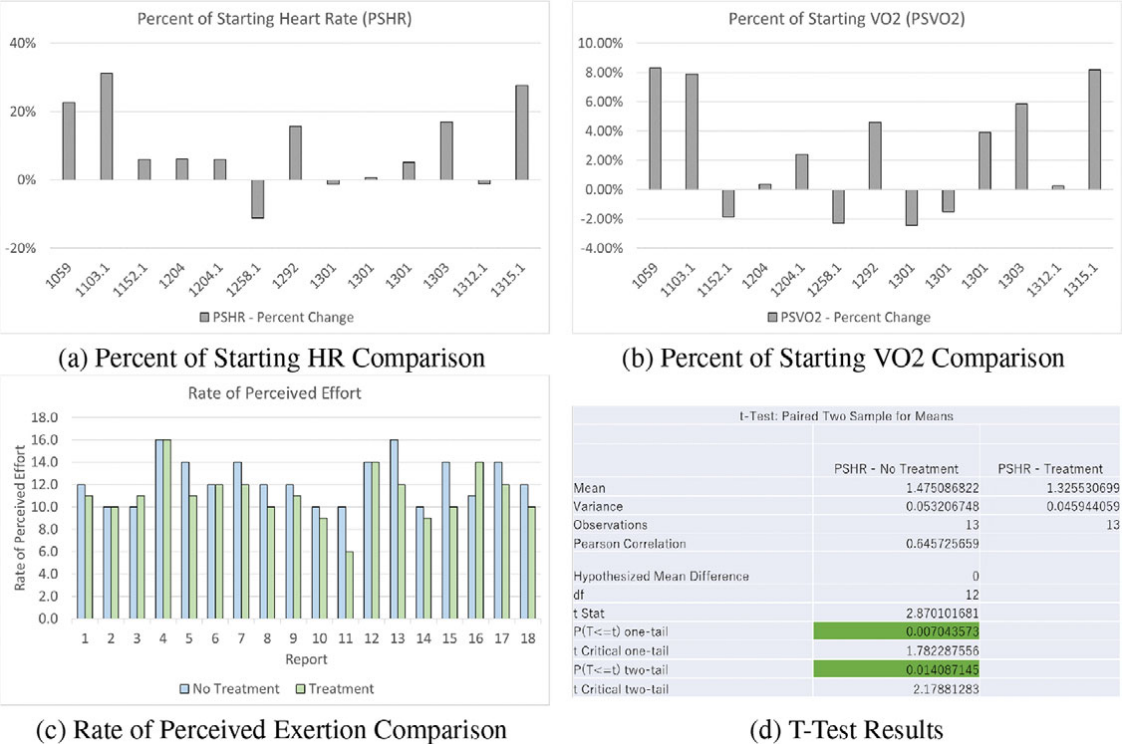
Graphs comparing percent of starting heart rate (a) and VO2 consumption (b). The rate of perceived exertion between APEx worn (green) and not worn (blue) is seen in (c). The results of the *t* test are shown in (d).

When compared (*n* = 13), the exoskeleton on configuration reduces the percents of starting heart rate and VO2 consumption (Figure 5a,b). On average, heart rate measured as beats per minute (BPM) was reduced by 13% and peak BPM by 18%. The percent of starting VO2 saw a peak reduction of about 8%. The APEx reduced the RPE by 8% (12.4 unequipped and 11.4 equipped) (Figure 5c). An additional *t* test (paired, two sample, confidence level 0.05, *n* = 13) was completed, showing that regardless of order, not wearing the exoskeleton resulted in an overall increased BPM (Figure 5d).

## Discussion

In a direct comparison and regardless of order, there are clear benefits to wearing the exoskeleton. The decreases in BPM, VO2, and RPE indicate that the exoskeleton in its current configuration is accomplishing the goals of the project. The test study was designed to have a lift task combined with a walking task. After talking with many users, we felt that this walking task was important to add because users will carry an object during palletizing. Mechanically, the APEX performed as expected. The catch and release mechanism allowed for uninhibited walking when carrying a load and activated at the base of lifts to provide assistance throughout. The strapping system conformed to the participant without interfering with their range of motion and causing undue stress on the body. Testing revealed a potential shortcoming in fitting, with the thigh paddle hitting higher on the thigh for taller individuals than as-tested on shorter individuals. It is possible that an adjustable mechanism or a longer paddle, granting a longer lever arm, provide a more comfortable fit and deliver stronger results.

## Conclusion and Future Work

The APEx was designed to be a compact, energy efficient powered exoskeleton to help aerial porters with lifting and pushing tasks. This was accomplished by using an innovative catch and release mechanism, a redundant control method, and lightweight materials. Though the APEx version tested is in a Beta state, it delivered significant reductions in all categories measured. Future iterations will further reduce the size and weight of the APEx by reducing the size of the electronics and altering the manufacturing materials. The materials used will also make for a more user-friendly exoskeleton, integrating all adjustable straps into a vest to reduce snag hazards. Ergonomists will also be consulted to improve fit on the wearer and mitigate potential future extended wear length issues. Further, the APEx will be tested to measure the efficacy in pushing tasks. It is hypothesized that, similar to lifting tasks, HR, VO2, and RPE will decrease when using the exoskeleton. The tests will also be completed in a lab that will allow for analysis of force and EMG signals.

## Data Availability

All available data will be furnished upon request to the corresponding authors.
